# Preoperative Systemic Inflammatory Indices and Their Association with Tumor Burden and Surgical Outcomes in High-Grade Serous Ovarian Cancer

**DOI:** 10.3390/diseases14040131

**Published:** 2026-04-03

**Authors:** Alexandru Marius Petrusan, Catalin Vladut Ionut Feier, Calin Muntean, Vasile Gaborean, Andrei Stefan Petrusan, Dragos Stefan Morariu, Ionut Flaviu Faur, Alaviana Monique Faur, Patriciu Achimas-Cadariu

**Affiliations:** 1 Doctoral School, “Iuliu Hațieganu” University of Medicine and Pharmacy, 400012 Cluj-Napoca, Romania; petrusan.alexandru.marius@elearn.umfcluj.ro; 2 Department of Surgical Oncology and Gynecologic Oncology, “Iuliu Hațieganu” University of Medicine and Pharmacy, 400012 Cluj-Napoca, Romania; pachimas@umfcluj.ro; 3 Department of Surgical Oncology, “Prof. Dr. I. Chiricuta” Institute of Oncology, 400015 Cluj-Napoca, Romania; dragosstefanmorariu@elearn.umfcluj.ro; 4 Abdominal Surgery and Phlebology Research Center, “Victor Babeş” University of Medicine and Pharmacy Timişoara, 300041 Timişoara, Romania; 5 First Surgery Clinic, “Pius Brinzeu” Clinical Emergency Hospital, 300723 Timişoara, Romania; 6 Medical Informatics and Biostatistics, Department III-Functional Sciences, “Victor Babeş” University of Medicine and Pharmacy Timişoara, Eftimie Murgu Square No. 2, 300041 Timişoara, Romania; 7 Thoracic Surgery Research Center, “Victor Babeş” University of Medicine and Pharmacy Timişoara, 300041 Timişoara, Romania; vasile.gaborean@umft.ro; 8 Department of Surgical Semiology, “Victor Babeş” University of Medicine and Pharmacy Timişoara, 300041 Timişoara, Romania; 9 Faculty of Medicine, “Iuliu Hațieganu” University of Medicine and Pharmacy, 400012 Cluj-Napoca, Romania; petrusan.andrei.stefan@elearn.umfcluj.ro; 10 IInd Surgery Clinic, Timisoara Emergency County Hospital, 300723 Timişoara, Romania; flaviu.faur@umft.ro; 11 X Department of General Surgery, “Victor Babeş” University of Medicine and Pharmacy Timişoara, 300041 Timişoara, Romania; 12 Department of Doctoral Studies, “Victor Babeş” University of Medicine and Pharmacy Timişoara, 300041 Timişoara, Romania; alaviana.faur@student.umft.ro

**Keywords:** high-grade serous ovarian cancer, systemic inflammatory markers, systemic immune-inflammation index, tumor burden, primary debulking surgery, perioperative outcomes, disease recurrence, prognostic biomarkers

## Abstract

Background/Objectives: High-grade serous ovarian cancer (HGSOC) represents the most aggressive subtype of epithelial ovarian cancer and is frequently diagnosed at advanced stages. Increasing evidence suggests that systemic inflammation plays an important role in tumor progression and clinical outcomes. This study aimed to evaluate the association between preoperative systemic inflammatory indices and tumor burden, perioperative outcomes, and recurrence risk in patients with HGSOC undergoing primary debulking surgery. Methods: We conducted a retrospective study including 125 patients with histopathologically confirmed HGSOC who underwent primary debulking surgery between January 2020 and December 2025. Preoperative hematological parameters obtained within 24 h before surgery were used to calculate inflammatory indices including the neutrophil-to-lymphocyte ratio (NLR), platelet-to-lymphocyte ratio (PLR), lymphocyte-to-monocyte ratio (LMR), systemic immune-inflammation index (SII), systemic inflammation response index (SIRI), and aggregate index of systemic inflammation (AISI). Associations between inflammatory markers, clinicopathological characteristics, perioperative outcomes, and recurrence were analyzed using non-parametric tests and logistic regression models. Results: The mean patient age was 53.66 ± 9.14 years, and most patients presented with advanced disease (FIGO III–IV: 70.4%). Patients with T3 tumors showed significantly higher monocyte (0.66 vs. 0.50 × 10^9^/L, *p* = 0.003), neutrophil (5.43 vs. 4.99 × 10^9^/L, *p* = 0.042), and platelet counts (325 vs. 280 × 10^9^/L, *p* = 0.006) and lower lymphocyte counts (1.79 vs. 1.96 × 10^9^/L, *p* = 0.009). Composite inflammatory indices were also increased in advanced disease, including PLR (177 vs. 153, *p* = 0.009), AISI (492 vs. 341, *p* = 0.002), and SIRI (1.65 vs. 1.18, *p* = 0.018). Patients requiring postoperative blood transfusion had higher neutrophil counts (7.65 vs. 4.97 × 10^9^/L, *p* < 0.001) and elevated SIRI (2.56 vs. 1.55, *p* < 0.001). Patients with recurrence had significantly higher platelet counts (339 vs. 293 × 10^9^/L, *p* = 0.001) and SII values (2849 vs. 2586, *p* = 0.012). In multivariate analysis, SII remained independently associated with recurrence (OR 1.022 per 100-unit increase; 95% CI 1.002–1.043; *p* = 0.033) together with advanced FIGO stages (OR 2.863; 95% CI 1.011–8.104; *p* = 0.048). Conclusions: Preoperative systemic inflammatory markers are significantly associated with tumor burden, surgical outcomes, and recurrence risk in HGSOC. An elevated SII appears to be an independent predictor of recurrence and may represent a practical biomarker for improving preoperative risk stratification and postoperative surveillance.

## 1. Introduction

Ovarian cancer remains one of the most lethal gynecologic malignancies worldwide, with recent global estimates reporting more than 300,000 new cases and over 200,000 related deaths each year [1]. Despite advances in surgical techniques and systemic therapies, it continues to represent the leading cause of mortality among cancers of the female reproductive tract. This unfavorable prognosis is largely explained by the absence of an effective population-based screening strategy and by the fact that most patients are diagnosed at advanced stages of disease [1,2]. Epidemiological studies consistently show that approximately 70–75% of women present with FIGO stage III or IV tumors at diagnosis, frequently associated with extensive transcoelomic spread throughout the peritoneal cavity [2,3]. Consequently, even in high-resource healthcare systems and in the context of modern therapeutic approaches, the five-year overall survival for advanced-stage ovarian cancer rarely exceeds 40–45% [1,4].

High-grade serous ovarian carcinoma (HGSOC) represents the predominant histological subtype of epithelial ovarian cancer, accounting for approximately 70% of cases and the majority of ovarian cancer–related deaths worldwide [5,6]. This tumor subtype is characterized by aggressive biological behavior, extensive intraperitoneal dissemination, and a strong tendency toward early relapse following primary treatment [6,7,8]. The current standard therapeutic strategy for advanced HGSOC consists of maximal cytoreductive surgery combined with platinum-based chemotherapy, with the goal of achieving complete macroscopic tumor resection whenever feasible [9]. Nevertheless, even when optimal cytoreduction and an initial response to platinum-based chemotherapy are achieved, the majority of patients experience disease recurrence within two to three years following primary treatment [6,10]. This high recurrence rate underscores the need to identify reliable biological markers capable of reflecting tumor aggressiveness and predicting clinical outcomes.

Growing evidence suggests that systemic inflammation plays a central role in cancer progression. Tumor-associated inflammatory responses contribute to several key mechanisms involved in carcinogenesis, including tumor proliferation, angiogenesis, immune escape, and metastatic dissemination [11,12]. In this context, increasing attention has been directed toward inflammation-based biomarkers derived from routine hematological parameters, as these markers may reflect the complex interaction between tumor biology and host immune response [13,14].

Among these markers, the neutrophil-to-lymphocyte ratio (NLR) has been widely investigated as an indicator of the balance between pro-tumor inflammatory activity and anti-tumor immune defense. Elevated neutrophil counts may reflect increased secretion of cytokines and growth factors that promote tumor progression, whereas decreased lymphocyte levels may indicate impaired cellular immune surveillance [13,15]. The platelet-to-lymphocyte ratio (PLR) has also been associated with tumor progression, as activated platelets release angiogenic mediators that support tumor growth and facilitate metastatic spread [16].

The lymphocyte-to-monocyte ratio (LMR) has been proposed as another marker reflecting the interaction between host immune response and tumor-associated macrophage activity. Circulating monocytes may differentiate into tumor-associated macrophages that promote angiogenesis and immunosuppression within the tumor microenvironment, whereas lymphocytes play a crucial role in anti-tumor immunity [17].

More recently, composite inflammatory indices integrating multiple hematological parameters have been developed to provide a more comprehensive assessment of systemic inflammatory status. The systemic immune-inflammation index (SII), calculated using neutrophil, platelet, and lymphocyte counts, has emerged as a promising prognostic biomarker reflecting both inflammatory activation and immune competence [18]. Similarly, the systemic inflammation response index (SIRI), which incorporates neutrophil, monocyte, and lymphocyte counts, has been proposed as a marker of the interaction between inflammatory pathways and immune regulation [19]. The aggregate index of systemic inflammation (AISI), combining neutrophils, monocytes, platelets, and lymphocytes, has also been suggested as a comprehensive indicator of cancer-related inflammatory burden [20].

Although these inflammatory indices have shown prognostic value in multiple malignancies, their clinical significance in high-grade serous ovarian carcinoma remains incompletely defined. Considering the aggressive biological behavior of HGSOC and the high rate of disease recurrence following primary treatment, the identification of simple and accessible biomarkers capable of reflecting tumor biology and predicting clinical outcomes is of particular interest. Therefore, the present study aimed to evaluate the relationship between preoperative systemic inflammatory markers and clinicopathological characteristics, as well as their potential role in predicting disease recurrence in patients undergoing primary debulking surgery for HGSOC.

## 2. Materials and Methods

This retrospective study analyzed the medical records of patients diagnosed with HGSOC who underwent primary debulking surgery at the “Prof. Dr. Ion Chiricuță” Institute of Oncology in Cluj-Napoca, Romania. Clinical and pathological data collected between 1 January 2020, and 31 December 2025, were reviewed.

Patients were included in the study if they met the following criteria:

- Histopathological confirmation of HGSOC on the postoperative specimen;
- Primary debulking surgery performed as the initial therapeutic approach;
- Treatment decision established within the institutional multidisciplinary tumor board based on preoperative imaging evaluation;
- Availability of complete clinical and laboratory data, including preoperative hematological parameters required for the calculation of inflammatory indices;
- Availability of tumor tissue and/or peripheral blood samples for BRCA mutation testing.

Patients who received neoadjuvant chemotherapy were excluded, as systemic treatment may alter inflammatory parameters and confound the assessment of preoperative inflammatory markers [21]. Additionally, patients with autoimmune diseases, prior malignancies, or synchronous cancers were excluded to minimize confounding related to chronic or tumor-associated systemic inflammation.

After applying the predefined inclusion criteria, a total of 125 patients were eligible for the study and included in the statistical analysis. Demographic data, including age and place of residence (urban versus rural), were recorded. Preoperative hematological parameters obtained from the complete blood count (obtained within 24 h prior to surgery) were collected and analyzed, including:

- Lymphocytes (Lym)
- Monocytes (Mon)
- Neutrophils (Neu)
- Platelets (Pla)

Based on these values, several inflammatory indices were calculated:

- NLR (Neutrophil-to-Lymphocyte Ratio) = Neu/Lym
- LMR (Lymphocyte-to-Monocyte Ratio) = Lym/Mon
- PLR (Platelet-to-Lymphocyte Ratio) = Pla/Lym
- AISI (Aggregate Index of Systemic Inflammation) = (Neu × Mon × Pla)/Lym
- SIRI (Systemic Inflammation Response Index) = (Mon × Neu)/Lym
- SII (Systemic Immune-Inflammation Index) = (Neu × Pla)/Lym

In addition to demographic and laboratory parameters, several clinicopathological variables were analyzed. These included FIGO stage, tumor invasion (T stage), and the presence of BRCA mutation, as well as vascular and lymphatic invasion identified on histopathological examination. All surgical procedures were performed with the aim of achieving maximal cytoreduction. Surgical and perioperative variables were also evaluated, including duration of surgery. Postoperative management variables, such as the need for intensive care unit (ICU) monitoring and the requirement for postoperative blood transfusion, were recorded. Furthermore, oncological outcomes were assessed by documenting residual disease status (R0 vs. R1 resection margins). Recurrence was defined as disease relapse within 24 months after surgery. To ensure uniform follow-up, only patients with at least 24 months of postoperative follow-up were included in this analysis. Accordingly, patients were required to have undergone surgery and been discharged no later than 31 December 2023.

The study protocol was approved by the Institutional Ethics Committee of the “Prof. Dr. Ion Chiricuță” Institute of Oncology (approval no. 371/24 February 2026). The study was conducted in accordance with the Declaration of Helsinki.

### Statistical Analysis

Statistical analysis was performed using IBM SPSS Statistics version 25 for Windows (IBM Corp., Armonk, NY, USA). The distribution of continuous variables was assessed using the Shapiro–Wilk test. Continuous variables were reported as mean ± standard deviation (SD) for normally distributed data and as median with interquartile range (IQR) for non-normally distributed variables. Categorical variables were expressed as frequencies and percentages.

Comparisons between two independent groups were performed using Student’s *t*-test for normally distributed variables and the Mann–Whitney U test for variables with non-normal distribution. Associations between categorical variables were evaluated using the Chi-square test or Fisher’s exact test, as appropriate.

To identify factors associated with disease recurrence, univariate logistic regression analysis was initially performed for clinically relevant variables. Variables showing potential association were subsequently included in a multivariate logistic regression model to determine independent predictors of recurrence. Because the systemic immune-inflammation index (SII) was expressed on a large numerical scale, the variable was entered into the regression models as SII/100 to facilitate interpretation of the odds ratios.

Results of the logistic regression analyses were reported as odds ratios (OR) with 95% confidence intervals (CI). A *p*-value < 0.05 was considered statistically significant for all analyses.

We utilized ChatGPT 5.2 an AI language model developed by OpenAI (San Francisco, CA, USA), to exclusively improve the manuscript’s language and readability. All the scientific content, interpretations, and conclusions are the original work of the authors

## 3. Results

A total of 125 patients who underwent primary debulking for HGSOC at the “Prof. Dr. Ion Chiricuță” Institute of Oncology in Cluj-Napoca were included in this study between 1 January 2020 and 31 December 2025.

### 3.1. Patient Characteristics and Perioperative Outcomes

The mean age of the patients was 53.66 ± 9.14 years, with the majority originating from urban areas (83 patients (66.4%) vs. 42 patients (33.6%)). Patients from urban areas were significantly older compared with those from rural areas (54.86 ± 8.69 vs. 51.29 ± 9.65 years, *p* = 0.039). BRCA mutation status was reported in 62 patients (49.6%). Among these, 40 cases (64.5%) were identified through tumor tissue testing and 22 (35.5%) through peripheral blood analysis.

Most patients were diagnosed with FIGO stage III disease (76 patients, 60.8%). The distribution of the FIGO stage and tumor invasion (T stage) as well as vascular and lymphatic invasion is presented in Table 1.

The mean duration of surgery was 100.56 ± 40.53 min. Regarding postoperative care, the majority of patients required monitoring in the intensive care unit (ICU) (80 patients, 64%), and 24 patients (19.2%) required postoperative blood transfusions.

Histopathological examination following surgery revealed positive resection margins (R1) in 20 cases (16%). Disease recurrence within two years after surgery was reported in 40 patients (32%).

### 3.2. Systemic Inflammatory Markers According to Tumor Characteristics and Perioperative Outcomes

When analyzed according to tumor invasion (T stage), patients with T3 tumors exhibited a more pronounced systemic inflammatory profile compared with those with T1–T2 tumors. Specifically, monocyte, neutrophil, and platelet counts were significantly higher in the T3 group, while lymphocyte counts and the LMR were lower. In addition, several composite inflammatory indices, including PLR, AISI, and SIRI, were significantly increased in patients with T3 tumors. In contrast, the NLR and SII did not differ significantly between the two groups. The detailed distribution of inflammatory markers according to T stage is presented in Table 2.

When stratified according to FIGO stage, patients with advanced disease (FIGO III–IV) exhibited a more pronounced systemic inflammatory profile compared with those with early-stage disease (FIGO I–II). Specifically, monocyte and platelet counts were higher in patients with FIGO III disease, while the LMR was lower, and several composite inflammatory indices, including the PLR, AISI, SIRI, and SII, were significantly increased in the advanced-stage group. The detailed distribution of inflammatory markers according to FIGO stage is presented in Table 3.

The association between systemic inflammatory markers and vascular invasion was also evaluated. Among the analyzed parameters, AISI values were significantly higher in patients with vascular invasion compared with those without vascular invasion (*p* = 0.036), suggesting a higher level of systemic inflammatory activation in this subgroup. In contrast, lymphocyte, monocyte, neutrophil, and platelet counts, as well as the remaining composite inflammatory indices (NLR, LMR, PLR, SIRI, and SII), did not differ significantly between the two groups.

The association between lymphatic invasion and systemic inflammatory markers was further explored. Patients with lymphatic invasion presented higher platelet counts (*p* = 0.023) and significantly increased values of several composite inflammatory indices, including the NLR (*p* = 0.043), PLR (*p* = 0.037), and SII (*p* = 0.016). In contrast, lymphocyte, monocyte, and neutrophil counts, as well as LMR, AISI, and SIRI values, were comparable between the two groups, with no statistically significant differences observed.

Patients who required postoperative blood transfusion showed higher levels of systemic inflammation compared with those who did not require transfusion. In particular, monocyte and neutrophil counts were significantly higher, while lymphocyte counts and LMRs were lower in the transfusion group. Composite inflammatory indices, including NLR, AISI, and SIRI, were also significantly increased. The platelet count, PLR, and SII did not differ significantly between the two groups (Table 4).

Patients requiring postoperative ICU monitoring showed higher levels of systemic inflammation compared with those managed outside the ICU. In particular, monocyte and neutrophil counts were significantly higher in patients admitted to the ICU, and composite inflammatory indices such as AISI and SIRI were also increased. The lymphocyte counts, platelet levels, NLR, LMR, PLR, and SII did not differ significantly between groups. The detailed distribution of inflammatory markers according to ICU admission is presented in Table 5.

The association between systemic inflammatory markers and residual disease status was also evaluated. Patients with residual disease after surgery (R1) exhibited significantly higher neutrophil counts (*p* = 0.001) and platelet levels (*p* < 0.001) compared with those who achieved complete cytoreduction (R0). In addition, several composite inflammatory indices, including PLR (*p* = 0.035), AISI (*p* = 0.044), and SII (*p* = 0.005), were significantly increased in the R1 group. In contrast, lymphocyte and monocyte counts, as well as the NLR, LMR, and SIRI, did not differ significantly between the two groups.

### 3.3. Inflammatory Markers and Predictors of Disease Recurrence

The potential association between systemic inflammatory markers and disease recurrence was further investigated. Patients were stratified according to the occurrence of recurrence during follow-up, and preoperative inflammatory markers were compared between the two groups. Among the analyzed parameters, differences were mainly observed in platelet counts and SIIs, while the remaining hematological parameters and composite inflammatory indices showed comparable distributions. The detailed values for each marker according to recurrence status are presented in Table 6.

To identify factors associated with recurrence, univariate logistic regression analysis was performed for clinically relevant variables, including age, FIGO stage, residual disease status, and the systemic immune-inflammation index (SII). Although platelet count showed a significant difference between groups in descriptive analysis, it was not included in the regression model, as it is incorporated into the SII and inclusion of both variables could introduce collinearity. Because SII values were expressed on a large numerical scale, the variable was entered into the model as SII/100 to facilitate interpretation of the odds ratio. Age was not associated with disease recurrence (OR 1.00, 95% CI 0.96–1.04, *p* = 1.000).

In contrast, advanced FIGO stage (III–IV) was significantly associated with disease recurrence, with patients presenting approximately 3.8-fold higher odds compared with those in stages I–II (OR 3.78, 95% CI 1.40–10.18, *p* = 0.009).

Residual disease after surgery was also significantly associated with this outcome, corresponding to an approximately 3.6-fold increase in the odds compared with patients who achieved complete cytoreduction (R0) (OR 3.56, 95% CI 1.18–10.69, *p* = 0.024).

Higher SII values were also associated with disease recurrence, corresponding to an approximately 2.5% increase in the odds for each 100-unit increase in the SII (OR 1.025, 95% CI 1.006–1.045, *p* = 0.010).

A multivariate logistic regression model was performed including age, FIGO stage, residual disease status (R0/R1), and the systemic immune-inflammation index (SII). In this model, higher SII values remained independently associated with disease recurrence, corresponding to an approximately 2.2% increase in the odds of recurrence for each 100-unit increase in SIIs. In addition, advanced FIGO stage (III–IV) was independently associated with recurrence, with patients presenting approximately 2.9-fold higher odds of recurrence compared with those in stages I–II. Age and residual disease status were not independently associated with recurrence in the multivariate analysis. The complete results of the multivariate model are presented in Table 7.

## 4. Discussion

### 4.1. Systemic Inflammatory Markers and Tumor Biology

This study examined the association between preoperative systemic inflammatory markers and tumor characteristics, perioperative outcomes, and recurrence among patients with HGSOC treated with primary debulking surgery. The findings indicate that systemic inflammatory indices correlate with tumor burden, surgical complexity, postoperative outcomes, and recurrence risk. In particular, patients presenting with extensive tumor invasion (T3) or advanced FIGO stage exhibited a more pronounced inflammatory profile, while elevated SII values remained independently associated with disease recurrence in multivariable analysis.

In our cohort of 125 patients, most cases were diagnosed at advanced stages, with 61.6% presenting FIGO stage III and 10.4% stage IV. This distribution reflects the well-recognized clinical pattern of epithelial ovarian cancer, which is frequently diagnosed late due to nonspecific symptoms and the absence of effective screening strategies. Epidemiological studies consistently report that approximately two-thirds of ovarian cancers are diagnosed at advanced stages, a factor strongly associated with poorer prognosis and extensive intraperitoneal dissemination [1,3,10].

One of the most notable findings of our study is the association between systemic inflammatory markers and tumor invasion. Patients with T3 tumors had significantly higher monocyte (*p* = 0.003), neutrophil (*p* = 0.042), and platelet counts (*p* = 0.006), together with lower lymphocyte (*p* = 0.009) levels and reduced LMR (*p* = 0.01) values. These results suggest that progressive tumor invasion is accompanied by systemic immune dysregulation characterized by increased innate immune activation and impaired adaptive immune responses. Neutrophils and monocytes may promote tumor progression through the secretion of cytokines, proteases, and pro-angiogenic mediators, whereas thrombocytosis can enhance tumor cell survival and metastatic dissemination. Conversely, lymphopenia may reflect reduced antitumor immune surveillance, contributing to an environment that favors tumor expansion [11,12,15].

Composite inflammatory indices further reinforced these observations. PLR, AISI, and SIRI were significantly higher in patients with T3 tumors (PLR *p* = 0.009; AISI *p* = 0.002; SIRI *p* = 0.018), indicating that integrated inflammatory markers may provide a more comprehensive representation of host–tumor interactions than individual hematological parameters. By simultaneously integrating neutrophil, lymphocyte, monocyte, and platelet counts, such composite markers capture the dynamic balance between tumor-promoting inflammatory processes and antitumor immune surveillance. Increasing evidence suggests that systemic inflammatory activation contributes directly to tumor progression through mechanisms such as cytokine-mediated signaling, promotion of angiogenesis, and facilitation of tumor cell invasion and dissemination [11,12,22]. In epithelial ovarian cancer, several studies have demonstrated that elevated inflammatory indices—including NLR, PLR, and SII—are associated with advanced tumor stage, higher tumor burden, and poorer clinical outcomes. Consequently, these composite biomarkers are increasingly considered promising and easily accessible tools for assessing tumor aggressiveness and improving risk stratification in patients with ovarian malignancies [23,24,25].

A similar pattern was observed when patients were stratified according to FIGO stage. Individuals with advanced disease (FIGO III–IV) displayed higher monocyte and platelet counts and lower LMR values compared with those with early-stage tumors. Composite indices including the PLR, AISI, SIRI, and SII were also markedly elevated in the advanced-stage group. These findings support the concept that systemic inflammatory activation reflects not only host immune responses but also the biological behavior of the tumor. In ovarian cancer, platelet-related indices have been associated with extensive peritoneal dissemination and increased tumor load, processes partly driven by tumor-derived cytokines that stimulate thrombopoiesis and myeloid cell recruitment. These mechanisms may promote angiogenesis and facilitate metastatic implantation within the peritoneal cavity [16,20,25].

Interestingly, although NLR is one of the most widely investigated inflammatory markers in oncology, it did not differ significantly between early and advanced tumor invasion in our cohort (*p* = 0.058). This observation is consistent with the heterogeneous findings reported in the literature. While several studies have reported strong prognostic significance for NLR in solid malignancies, others have not confirmed its independent predictive value in ovarian cancer populations. Differences in study design, patient characteristics, tumor biology, and cut-off thresholds may partly explain these inconsistencies [13,14].

### 4.2. Systemic Inflammation and Perioperative Surgical Outcomes

Our analysis also demonstrated significant associations between systemic inflammatory markers and perioperative outcomes. Patients requiring postoperative blood transfusion exhibited higher monocyte and neutrophil counts together with lower lymphocyte levels and reduced LMR values. In addition, composite indices such as the NLR, AISI, and SIRI were significantly elevated in this group (NLR *p* = 0.009; AISI *p* = 0.001; SIRI *p* < 0.001). These findings suggest that heightened systemic inflammatory activation may be associated with more extensive surgical procedures or greater tumor burden, both of which may contribute to increased intraoperative blood loss [8,9].

Cytoreductive surgery for advanced ovarian cancer often involves complex procedures, including multiorgan resections and peritonectomy techniques, which are associated with prolonged operative time and increased surgical morbidity [8,9]. Previous studies have demonstrated that surgical radicality remains a key determinant of perioperative outcomes when maximal cytoreduction is pursued. In this context, systemic inflammatory activation may reflect biologically aggressive disease requiring technically demanding surgical management [26,27].

Inflammatory mediators released by tumor and immune cells may further influence vascular integrity and coagulation pathways. Cytokine-driven activation of myeloid cells and platelets can alter endothelial function and microvascular stability, potentially increasing the risk of bleeding during major oncologic surgery. Consequently, elevated inflammatory indices may indirectly reflect both tumor biology and the anticipated complexity of surgical management in patients undergoing primary debulking procedures [12,15,16].

A similar pattern was observed when perioperative intensive care requirements were analyzed. Patients requiring postoperative ICU monitoring presented significantly higher neutrophil and monocyte counts together with elevated AISI and SIRI values, suggesting that preoperative inflammatory activation may also influence early postoperative physiological resilience. Several studies have suggested that systemic inflammatory indices may serve as indicators of the host response to surgical stress, with elevated preoperative values being associated with increased rates of postoperative complications following major oncologic procedures [12,28,29].

The interaction between inflammatory activation and tumor dissemination was further highlighted by the association between lymphatic invasion and elevated platelet-related indices observed in our cohort. Patients with lymphatic invasion demonstrated significantly higher platelet counts together with increased NLR, PLR, and SII values. These findings are consistent with growing evidence indicating that platelets actively participate in metastatic processes by facilitating tumor cell survival within the circulation and promoting adhesion to vascular and lymphatic endothelium [16,30]. Through the release of growth factors and chemokines, activated platelets may also stimulate lymphangiogenesis and enhance the ability of malignant cells to colonize distant tissues [11,31].

The relationship between inflammatory markers and residual disease further supports this concept. Patients with residual tumor following cytoreductive surgery exhibited higher neutrophil and platelet counts together with increased PLR, AISI, and SII values. These findings suggest that tumors characterized by pronounced inflammatory activity may display more diffuse peritoneal spread, potentially limiting the feasibility of complete cytoreduction. Similar associations between inflammatory biomarkers and suboptimal cytoreduction have been reported in previous studies [15,32].

### 4.3. Systemic Inflammation and Risk of Disease Recurrence

Beyond perioperative outcomes, our results demonstrate a significant association between systemic inflammatory markers and recurrence risk. Patients who developed recurrence within two years exhibited significantly higher platelet counts and elevated SII values compared with those who remained disease-free. These findings suggest that systemic inflammatory activation may contribute to tumor persistence and early relapse following cytoreductive surgery.

The prognostic role of the SII was further supported by regression analyses. In univariate analysis, increasing SII values were significantly associated with recurrence risk, and this association remained independent in the multivariable model (OR 1.022 per 100-unit increase (95% CI 1.002–1.043, *p* = 0.033)) together with the FIGO stage. These results indicate that systemic inflammatory status may capture biological information not fully reflected by traditional clinicopathological parameters. Previous studies have similarly demonstrated that elevated SII levels are associated with poorer progression-free and overall survival in ovarian cancer patients [33,34].

Several biological mechanisms may explain this relationship. Neutrophils and monocytes are known to contribute to tumor progression through the release of pro-angiogenic mediators, matrix-degrading enzymes, and immunosuppressive cytokines that facilitate tumor cell invasion and metastatic niche formation. In parallel, platelets can promote tumor cell survival during hematogenous dissemination and enhance metastatic colonization by protecting circulating tumor cells from immune-mediated destruction and by supporting endothelial adhesion [30,35]. Conversely, reduced lymphocyte levels may indicate impaired antitumor immune surveillance, limiting the host’s capacity to eliminate residual malignant cells following surgery and systemic therapy.

Because the SII integrates neutrophil, platelet, and lymphocyte counts into a single index, it provides a comprehensive representation of the interaction between tumor-promoting inflammation and host immune defense mechanisms. Elevated preoperative SII values may therefore identify patients with a biologically permissive inflammatory environment that favors tumor persistence and early recurrence. These findings support the potential use of systemic inflammatory indices as accessible biomarkers for identifying patients at increased risk of relapse and for refining postoperative surveillance strategies in epithelial ovarian cancer [33,34,36].

The clinical implications of these findings are significant. Systemic inflammatory markers are inexpensive, widely available, and easily measurable through routine blood tests. Their incorporation into clinical practice may improve preoperative risk stratification and help identify patients with more aggressive tumor biology who could benefit from closer postoperative monitoring or more intensive systemic therapy.

### 4.4. Study Limitations

Several limitations should be acknowledged. The retrospective design and single-center setting may limit the generalizability of the results. In addition, surgical procedures were performed by multiple surgeons and histopathological evaluation involved several pathologists, which may have introduced variability in surgical decision-making and pathological assessment. The study period also partially overlapped with the COVID-19 pandemic, during which delays in diagnosis and treatment have been reported, potentially resulting in a higher proportion of patients presenting with advanced disease stages [37,38,39].

Furthermore, systemic inflammatory indices were derived from a single preoperative blood sample obtained within 24 h prior to surgery. Although this approach reflects common clinical practice and captures the inflammatory status at a clinically relevant time point, it may not fully account for temporal variations and potential transient influences. In addition, given the retrospective design, the observed associations should be interpreted with caution, as inflammatory markers may partly reflect underlying tumor burden and disease characteristics and given the multiple comparisons performed, the possibility of type I error should be considered when interpreting the results.

Finally, although the cohort was representative for patients undergoing primary debulking surgery, the relatively limited sample size may have reduced the statistical power of certain analyses.

Despite these limitations, the present study provides further evidence supporting the clinical relevance of systemic inflammatory markers in ovarian cancer, particularly in relation to tumor burden and perioperative outcomes. Future prospective multicenter studies are warranted to further validate these findings and to better define their prognostic role.

## 5. Conclusions

Preoperative systemic inflammatory markers are significantly associated with tumor burden, disease stage, perioperative outcomes, and recurrence risk in patients with high-grade serous ovarian cancer undergoing primary debulking surgery. In particular, elevated systemic immune-inflammation index (SII) values were independently associated with disease recurrence, suggesting that systemic inflammation reflects both tumor aggressiveness and host–tumor interactions. Given their availability through routine laboratory testing, inflammatory indices may represent practical biomarkers for improving preoperative risk stratification and postoperative surveillance in ovarian cancer. Further prospective multicenter studies are needed to validate these findings and to clarify their role in personalized clinical management.

## Figures and Tables

**Table 1:** Distribution of tumor stage and invasion characteristics in the study population.

Variables	All (n = 125)
T1	22 (17.6%)
T2	19 (15.2%)
T3	84 (67.2%)
FIGO stage	
I	18 (14.4%)
II	17 (13.6%)
III	77 (61.6%)
IV	13 (10.4%)
Lymphatic invasion	54 (43.2%)
Vascular invasion	18 (14.4%)

**Table 2:** Distribution of systemic inflammatory markers according to tumor invasion (T stage).

Marker	All, n = 125	T1–T2, n = 41	T3, n = 84	*p*
Lymphocytes (×0^9^/L)	1.83 [1.35–2.23]	1.96 [1.58–2.58]	1.79 [1.32–2.19]	0.009
Monocytes (×10^9^/L)	0.61 [0.52–0.73]	0.50 [0.46–0.70]	0.66 [0.55–0.79]	0.003
Neutrophils (×10^9^/L)	5.29 [3.94–6.79]	4.99 [3.95–5.50]	5.43 [3.94–7.53]	0.042
Platelets (×10^9^/L)	311 [276–383]	280 [243–331]	325 [288–405]	0.006
NLR	2.94 [2.02–3.92]	2.44 [1.94–3.60]	3.19 [2.06–4.01]	0.058
LMR	3.31 [2.32–4.24]	3.46 [3.00–4.80]	2.99 [2.12–3.87]	0.01
PLR	169 [135–248]	153 [110–179]	177 [141–277]	0.009
AISI	442 [276–846]	341 [232–477]	492 [292–1000]	0.002
SIRI	1.49 [0.98–2.71]	1.18 [0.81–1.77]	1.65 [1.13–3.31]	0.018
SII	2693 [2152–3663]	2579 [1776–3143]	2731 [2265–3974]	0.087

Data are reported as median [Q1 to Q3], where Q is the quartile. Mann–Whitney test was used to compare the two independent groups.

**Table 3:** Systemic inflammatory markers according to FIGO stage.

Marker	All, n = 125	Figo I–II, n = 35	Figo III–IV, n = 90	*p*
Lymphocytes (×10^9^/L)	1.83 [1.35–2.23]	1.92 [1.45–2.56]	1.81 [1.36–2.23]	0.161
Monocytes (×10^9^/L)	0.61 [0.52–0.73]	0.49 [0.47–0.70]	0.65 [0.55–0.78]	0.027
Neutrophils (×10^9^/L)	5.29 [3.94–6.79]	4.96 [3.96–5.50]	5.38 [3.93–7.52]	0.098
Platelets (×10^9^/L)	311 [276–383]	268 [241–327]	325 [290–402]	0.002
NLR	2.94 [2.02–3.92]	2.56 [1.62–3.60]	2.98 [2.08–4.01]	0.102
LMR	3.31 [2.32–4.24]	3.58 [3.26–4.77]	3.00 [2.22–3.87]	0.019
PLR	169 [135–248]	157 [101–179]	166 [140–249]	0.013
AISI	442 [276–846]	341 [226–475]	492 [289–916]	<0.001
SIRI	1.49 [0.98–2.71]	1.18 [0.81–1.65]	1.65 [1.13–2.66]	0.021
SII	2693 [2152–3663]	2579 [1758–3135]	2731 [2279–4012]	0.042

Data are reported as median [Q1 to Q3], where Q is the quartile. Mann–Whitney test was used to compare the two independent groups.

**Table 4:** Systemic inflammatory markers according to necessity of postoperative blood transfusion.

Marker	All, n = 125	No Blood Transfusion, n = 101	Blood Transfusion, n = 24	*p*
Lymphocytes (×10^9^/L)	1.83 [1.35–2.23]	1.81 [1.40–2.39]	1.66 [1.35–1.90]	0.046
Monocytes (×10^9^/L)	0.61 [0.52–0.73]	0.57 [0.48–0.73]	0.76 [0.65–0.82]	0.001
Neutrophils (×10^9^/L)	5.29 [3.94–6.79]	4.97 [3.91–6.09]	7.65 [5.77–8.90]	<0.001
Platelets (×10^9^/L)	311 [276–383]	314 [267–357]	312 [259–430]	0.763
NLR	2.94 [2.02–3.92]	2.60 [2.04–3.66]	3.63 [2.40–6.93]	0.009
LMR	3.31 [2.32–4.24]	3.26 [2.73–4.41]	2.43 [1.23–3.26]	0.004
PLR	169 [135–248]	160 [122–223]	186 [149–362]	0.072
AISI	442 [276–846]	475 [237–623]	796 [452–2437]	0.001
SIRI	1.49 [0.98–2.71]	1.55 [0.86–1.84]	2.56 [1.65–6.29]	<0.001
SII	2693 [2152–3663]	2651 [1938–3561]	2852 [2173–4857]	0.179

Data are reported as median [Q1 to Q3], where Q is the quartile. Mann–Whitney test was used to compare the two independent groups.

**Table 5:** Systemic inflammatory markers according to postoperative ICU admission.

Marker	All, n = 125	No ICU Admission (n = 45)	ICU Admission (n = 80)	*p*
Lymphocytes (×10^9^/L)	1.83 [1.35–2.23]	1.79 [1.36–2.32]	1.83 [1.44–2.28]	0.765
Monocytes (×10^9^/L)	0.61 [0.52–0.73]	0.49 [0.45–0.61]	0.68 [0.56–0.82]	<0.001
Neutrophils (×10^9^/L)	5.29 [3.94–6.79]	4.49 [3.62–5.04]	5.88 [4.43–7.79]	<0.001
Platelets (×10^9^/L)	311 [276–383]	292 [268–336]	324 [260–425]	0.069
NLR	2.94 [2.02–3.92]	2.87 [2.07–3.60]	2.61 [2.05–4.64]	0.166
LMR	3.31 [2.32–4.24]	3.26 [2.73–4.27]	3.15 [1.96–4.19]	0.136
PLR	169 [135–248]	162 [143–210]	162 [121–262]	0.499
AISI	442 [276–846]	465 [237–504]	517 [267–1418]	0.018
SIRI	1.49 [0.98–2.71]	1.55 [0.86–1.76]	1.65 [1.12–3.96]	0.033
SII	2693 [2152–3663]	2586 [1865–3135]	2783 [1944–4093]	0.103

Data are reported as median [Q1 to Q3], where Q is the quartile. Mann–Whitney test was used to compare the two independent groups.

**Table 6:** Systemic inflammatory markers according to presence of recurrence.

Marker	All, n = 125	No Recurrence (n = 85)	Recurrence (n = 40)	*p*
Lymphocytes (×10^9^/L)	1.83 [1.35–2.23]	1.78 [1.40–2.40]	1.85 [1.49–2.11]	0.474
Monocytes (×10^9^/L)	0.61 [0.52–0.73]	0.61 [0.49–0.74]	0.60 [0.48–0.80]	0.903
Neutrophils (×10^9^/L)	5.29 [3.94–6.79]	5.06 [3.96–6.38]	4.78 [3.85–6.91]	0.618
Platelets (×10^9^/L)	311 [276–383]	293 [243–331]	339 [290–489]	0.001
NLR	2.94 [2.02–3.92]	2.61 [2.05–3.80]	3.43 [2.10–4.24]	0.392
LMR	3.31 [2.32–4.24]	3.26 [2.59–3.94]	2.78 [1.89–4.40]	0.640
PLR	169 [135–248]	162 [122–210]	178 [136–287]	0.261
AISI	442 [276–846]	475 [250–686]	510 [267–1308]	0.097
SIRI	1.49 [0.98–2.71]	1.55 [1.14–2.36]	1.67 [1.12–4.02]	0.356
SII	2693 [2152–3663]	2586 [1938–3138]	2849 [2570–4573]	0.012

Data are reported as median [Q1 to Q3], where Q is the quartile. Mann–Whitney test was used to compare the two independent groups.

**Table 7:** Multivariate logistic regression.

Variables in the Equation									
		B	S.E.	Wald	df	Sig.	Exp(B)	95% C.I. for EXP(B)	
								Lower	Upper
Step 1 ^a^	SII/100	0.022	0.010	4.545	1	0.033	1.022	1.002	1.043
	Age (years)	0.019	0.024	0.604	1	0.437	1.019	0.972	1.067
	R1	0.808	0.610	1.752	1	0.186	2.242	0.678	7.413
	FIGO I–II vs. III–IV	1.052	0.531	3.926	1	0.048	2.863	1.011	8.104
	Constant	−4.251	1.684	6.369	1	0.012	0.014		

^a^ Variable(s) entered on step 1: SII/100, Age (years), R1, FIGO I–II vs. III–IV.

## Data Availability

Data available on request due to patient confidentiality, privacy protection regulations (including GDPR), and institutional policies governing access to clinical data. Further inquiries can be made upon reasonable request from the corresponding authors.

## References

[1] Sung H., Ferlay J., Siegel R.L., Laversanne M., Soerjomataram I., Jemal A., Bray F. (2021). Global Cancer Statistics 2020: GLOBOCAN Estimates of Incidence and Mortality Worldwide for 36 Cancers in 185 Countries. CA Cancer J. Clin..

[2] Lheureux S., Gourley C., Vergote I., Oza A.M. (2019). Epithelial ovarian cancer. Lancet.

[3] Torre L.A., Trabert B., DeSantis C.E., Miller K.D., Samimi G., Runowicz C.D., Gaudet M.M., Jemal A., Siegel R.L. (2018). Ovarian cancer statistics, 2018. CA Cancer J. Clin..

[4] Siegel R.L., Miller K.D., Wagle N.S., Jemal A. (2023). Cancer statistics, 2023. CA Cancer J. Clin..

[5] Kurman R.J., Shih I.e.M. (2016). The Dualistic Model of Ovarian Carcinogenesis: Revisited, Revised, and Expanded. Am. J. Pathol..

[6] Bowtell D.D., Böhm S., Ahmed A.A., Aspuria P.-J., Bast R.C., Beral V., Berek J.S., Birrer M.J., Blagden S., Bookman M.A. (2015). Rethinking ovarian cancer II: Reducing mortality from high-grade serous ovarian cancer. Nat. Rev. Cancer.

[7] Vaughan S., Coward J.I., Bast R.C., Berchuck A., Berek J.S., Brenton J.D., Coukos G., Crum C.C., Drapkin R., Etemadmoghadam D. (2011). Rethinking ovarian cancer: Recommendations for improving outcomes. Nat. Rev. Cancer.

[8] du Bois A., Reuss A., Pujade-Lauraine E., Harter P., Ray-Coquard I., Pfisterer J. (2009). Role of surgical outcome as prognostic factor in advanced epithelial ovarian cancer: A combined exploratory analysis of 3 prospectively randomized phase 3 multicenter trials: By the Arbeitsgemeinschaft Gynaekologische Onkologie Studiengruppe Ovarialkarzinom (AGO-OVAR) and the Groupe d’Investigateurs Nationaux Pour les Etudes des Cancers de l’Ovaire (GINECO). Cancer.

[9] Bristow R.E., Tomacruz R.S., Armstrong D.K., Trimble E.L., Montz F.J. (2002). Survival effect of maximal cytoreductive surgery for advanced ovarian carcinoma during the platinum era: A meta-analysis. J. Clin. Oncol..

[10] Lheureux S., Braunstein M., Oza A.M. (2019). Epithelial ovarian cancer: Evolution of management in the era of precision medicine. CA Cancer J. Clin..

[11] Hanahan D., Weinberg R.A. (2011). Hallmarks of cancer: The next generation. Cell.

[12] Grivennikov S.I., Greten F.R., Karin M. (2010). Immunity, inflammation, and cancer. Cell.

[13] Templeton A.J., Mcnamara M.G., Šeruga B., Vera-Badillo F.E., Aneja P., Ocaña A., Leibowitz-Amit R., Sonpavde G., Knox J.J., Tran B. (2014). Prognostic role of neutrophil-to-lymphocyte ratio in solid tumors: A systematic review and meta-analysis. J. Natl. Cancer Inst..

[14] Guthrie G.J., Charles K.A., Roxburgh C.S., Horgan P.G., McMillan D.C., Clarke S.J. (2013). The systemic inflammation-based neutrophil-lymphocyte ratio: Experience in patients with cancer. Crit. Rev. Oncol. Hematol..

[15] Mantovani A., Allavena P., Sica A., Balkwill F. (2008). Cancer-related inflammation. Nature.

[16] Stone R.L., Nick A.M., McNeish I.A., Balkwill F., Han H.D., Bottsford-Miller J., Rupaimoole R., Armaiz-Pena G.N., Pecot C.V., Coward J. (2012). Paraneoplastic thrombocytosis in ovarian cancer. N. Engl. J. Med..

[17] Nishijima T.F., Muss H.B., Shachar S.S., Tamura K., Takamatsu Y. (2015). Prognostic value of lymphocyte-to-monocyte ratio in patients with solid tumors: A systematic review and meta-analysis. Cancer Treat. Rev..

[18] Hu B., Yang X.R., Xu Y., Sun Y.-F., Sun C., Guo W., Zhang X., Wang W.-M., Qiu S.-J., Zhou J. (2014). Systemic immune-inflammation index predicts prognosis of patients after curative resection for hepatocellular carcinoma. Clin. Cancer Res..

[19] Qi Q., Zhuang L., Shen Y., Geng Y., Yu S., Chen H., Liu L., Meng Z., Wang P., Chen Z. (2016). A novel systemic inflammation response index (SIRI) for predicting the survival of patients with pancreatic cancer after chemotherapy. Cancer.

[20] Asher V., Lee J., Innamaa A., Bali A. (2011). Preoperative platelet lymphocyte ratio as an independent prognostic marker in ovarian cancer. Clin. Transl. Oncol..

[21] Kehoe S., Hook J., Nankivell M., Jayson G.C., Kitchener H., Lopes T., Luesley D., Perren T., Bannoo S., Mascarenhas M. (2015). Primary chemotherapy versus primary surgery for newly diagnosed advanced ovarian cancer (CHORUS): An open-label, randomised, controlled, non-inferiority trial. Lancet.

[22] Coussens L.M., Werb Z. (2002). Inflammation and cancer. Nature.

[23] Dinca A.L., Diaconu A., Birla R.D., Coculescu B.-I., Dinca V.-G., Manole G., Marica C., Tudorache I.S., Panaitescu E., Constantinoiu S.M. (2023). Systemic inflammation factors as survival prognosis markers in ovarian neoplasm and the relationship with cancer-associated inflammatory mediators—A review. Int. J. Immunopathol. Pharmacol..

[24] Yang Y., Hu Z., Ye Y., Wu H., Sun W., Wang N. (2025). Association of aggregate index of systemic inflammation with increased all-cause and cardiovascular mortality in female cancer patients. Front. Oncol..

[25] Ohanoglu Cetinel K., Guner G., Karabudak C.B., Zeynalli E., Yavuz Sen S. (2026). Dynamic changes in prognostic nutritional index and systemic immune-inflammation index predict survival following debulking surgery in ovarian cancer. Front. Oncol..

[26] Aletti G.D., Dowdy S.C., Gostout B.S., Jones M.B., Stanhope C.R., Wilson T.O., Podratz K.C., Cliby W.A. (2006). Aggressive surgical effort and improved survival in advanced-stage ovarian cancer. Obstet. Gynecol..

[27] Chi D.S., Eisenhauer E.L., Zivanovic O., Sonoda Y., Abu-Rustum N.R., Levine D.A., Guile M.W., Bristow R.E., Aghajanian C., Barakat R.R. (2009). Improved progression-free and overall survival in advanced ovarian cancer as a result of a change in surgical paradigm. Gynecol. Oncol..

[28] Forget P., Khalifa C., Defour J.P., Latinne D., Van Pel M.C., De Kock M. (2017). What is the normal value of the neutrophil-to-lymphocyte ratio?. BMC Res. Notes.

[29] Raungkaewmanee S., Tangjitgamol S., Manusirivithaya S., Srijaipracharoen S., Thavaramara T. (2012). Platelet to lymphocyte ratio as a prognostic factor for epithelial ovarian cancer. J. Gynecol. Oncol..

[30] Labelle M., Begum S., Hynes R.O. (2011). Direct signaling between platelets and cancer cells induces an epithelial-mesenchymal-like transition and promotes metastasis. Cancer Cell.

[31] Gay L.J., Felding-Habermann B. (2011). Contribution of platelets to tumour metastasis. Nat. Rev. Cancer.

[32] Zhu Y., Zhou S., Liu Y., Zhai L., Sun X. (2018). Prognostic value of systemic inflammatory markers in ovarian Cancer: A PRISMA-compliant meta-analysis and systematic review. BMC Cancer.

[33] Chu B., Chen Y., Pan J. (2025). Prognostic significance of systemic immune inflammation index for ovarian cancer: An updated systematic review and meta-analysis. J. Ovarian Res..

[34] Chen J., Jin L., Luo R., Zhang X., Chen Y., Han Z., Liu T. (2025). Predictive value of preoperative systemic immune-inflammation index and prognostic nutrition index in patients with epithelial ovarian cancer. J. Ovarian Res..

[35] Mao H., Yang F. (2023). Prognostic significance of systemic immune-inflammation index in patients with ovarian cancer: A meta-analysis. Front. Oncol..

[36] El Bairi K., Al Jarroudi O., Afqir S. (2021). Inexpensive Systemic Inflammatory Biomarkers in Ovarian Cancer: An Umbrella Systematic Review of 17 Prognostic Meta-Analyses. Front. Oncol..

[37] Wang Y., Chang L., He H., Zhang X., Liu J., Zang L., Li L., Liu T., Xu Q. (2025). The impact of the COVID-19 pandemic on the diagnosis, treatment, and prognosis of ovarian cancer in the United States: A retrospective cohort study based on the SEER database. Discov. Oncol..

[38] Puia A., Pop S.R., Manzat B.O.C., Pintea S., Puia I.C., Fadgyas-Stanculete M. (2025). Coping Strategies Among Healthcare Workers During the COVID-19 Pandemic: Emotional Responses, Challenges, and Adaptive Practices. Medicina.

[39] Moterani V.C., Moterani N.J.W., Candido Dos Reis F.J. (2022). Treatment delay and treatment pattern modifications among epithelial ovarian cancer patients during the COVID-19 pandemic: A retrospective cohort study. J. Surg. Oncol..

